# Clinical value of anti-desmoglein-2 antibodies in arrhythmogenic right ventricular cardiomyopathy: real-world evidence

**DOI:** 10.1093/europace/euag130

**Published:** 2026-06-03

**Authors:** Shaylyn Joseph, Karolina Borowiec, Olgierd Woźniak, Diptendu Chatterjee, Meena Fatah, Vivian Silbiger, Ilona Kowalik, Urszula Skrzypczyńska-Banasik, Ewa Kowalik, Mirosław Kowalski, Elżbieta Katarzyna Biernacka, Robert M Hamilton

**Affiliations:** Department of Pediatrics, The Labatt Family Heart Centre and Translational Medicine, The Hospital for Sick Children & Research Institute and the University of Toronto, Room 1725D, 555 University Avenue, Toronto, ON M5G 1X8, Canada; Department of Congenital Heart Diseases, National Institute of Cardiology, 42 Alpejska Str., Warsaw 04-628, Poland; Department of Congenital Heart Diseases, National Institute of Cardiology, 42 Alpejska Str., Warsaw 04-628, Poland; Department of Pediatrics, The Labatt Family Heart Centre and Translational Medicine, The Hospital for Sick Children & Research Institute and the University of Toronto, Room 1725D, 555 University Avenue, Toronto, ON M5G 1X8, Canada; Department of Pediatrics, The Labatt Family Heart Centre and Translational Medicine, The Hospital for Sick Children & Research Institute and the University of Toronto, Room 1725D, 555 University Avenue, Toronto, ON M5G 1X8, Canada; Department of Pediatrics, The Labatt Family Heart Centre and Translational Medicine, The Hospital for Sick Children & Research Institute and the University of Toronto, Room 1725D, 555 University Avenue, Toronto, ON M5G 1X8, Canada; Clinical Research Support Centre, National Institute of Cardiology, 42 Alpejska Str., Warsaw 04-628, Poland; Department of Congenital Heart Diseases, National Institute of Cardiology, 42 Alpejska Str., Warsaw 04-628, Poland; Department of Congenital Heart Diseases, National Institute of Cardiology, 42 Alpejska Str., Warsaw 04-628, Poland; Department of Congenital Heart Diseases, National Institute of Cardiology, 42 Alpejska Str., Warsaw 04-628, Poland; Department of Congenital Heart Diseases, National Institute of Cardiology, 42 Alpejska Str., Warsaw 04-628, Poland; Department of Pediatrics, The Labatt Family Heart Centre and Translational Medicine, The Hospital for Sick Children & Research Institute and the University of Toronto, Room 1725D, 555 University Avenue, Toronto, ON M5G 1X8, Canada

**Keywords:** Arrhythmogenic right ventricular cardiomyopathy, Autoantibodies, Desmoglein-2 • Biomarker

## Introduction

Arrhythmogenic right ventricular cardiomyopathy (ARVC) is an inherited myocardial disease characterized by fibrofatty replacement of ventricular myocardium, predisposing affected individuals to life-threatening ventricular arrhythmias and sudden cardiac death.^1,2^ While classically a right ventricular disease, biventricular and left-dominant forms are increasingly recognized, broadening the diagnostic challenge. Despite advances in imaging and genetics, diagnosis remains challenging due to phenotypic heterogeneity, age-dependent expression, and incomplete genetic penetrance.^3,4^ Although pathogenic variants in desmosomal genes are a defining feature of ARVC, up to 40% of patients remain genetically elusive, limiting diagnostic yield.^5^ Current frameworks, including the 2010 Task Force Criteria and the Padua criteria, integrate imaging, electrocardiographic, arrhythmic, and genetic findings but remain insensitive in early or atypical presentations, underscoring the need for complementary biomarkers.^3,4^

Autoimmune mechanisms have been implicated in ARVC pathogenesis. Circulating autoantibodies directed against intercalated disc proteins, including desmoglein-2 (DSG2), have been identified in prior work and may contribute to disease progression by interfering with desmosomal function and promoting pro-inflammatory signalling.^5–8^ Anti-DSG2 antibodies appear largely absent in healthy individuals, suggesting disease specificity.^5^ However, their diagnostic and prognostic relevance in ARVC remains incompletely characterized. We therefore evaluated the utility of anti-DSG2 antibodies in a well-characterized ARVC cohort, with comparison to non-ARVC right ventricular disease and healthy controls.

## Methods

We studied 101 patients with a definite diagnosis of ARVC according to the 2010 Task Force Criteria,^3^ recruited at a single tertiary referral centre. Two control groups were included: 37 patients with right ventricular conditions unrelated to ARVC (Ebstein anomaly or Eisenmenger syndrome) and 31 healthy individuals. Serum samples were isolated from peripheral blood and stored at −80°C prior to analysis. Anti-DSG2 antibody levels were quantified by a standardized ELISA targeting extracellular domains 3 and 4 and the extracellular anchor region of DSG2, as previously described,^6^ with results expressed as optical density (OD). Patients were followed for cardiac death or heart transplantation (primary endpoint) and major arrhythmic events.

Group comparisons were performed using non-parametric testing. Diagnostic and prognostic cut-offs were derived using receiver operating characteristic (ROC) analysis with Youden index optimization. Survival analyses were conducted using Kaplan–Meier methods and Cox proportional hazards models.

## Results

The ARVC cohort included 101 patients [64 males (63.4%) and 37 females (36.6%)] with a mean age of 46.8 ± 15.8 years; 62.4% had a history of sustained ventricular tachycardia, 64.4% carried an implantable cardioverter-defibrillator (ICD), and left ventricular involvement (LVEF < 50%) was present in 30.7%. Genetic testing identified pathogenic variants in 59 of 101 patients (58%), most commonly in *PKP2* (48.5%), followed by *DSG2* (5%) and other ARVC-associated genes (5%); 35.6% were gene elusive.

**Figure 1:**
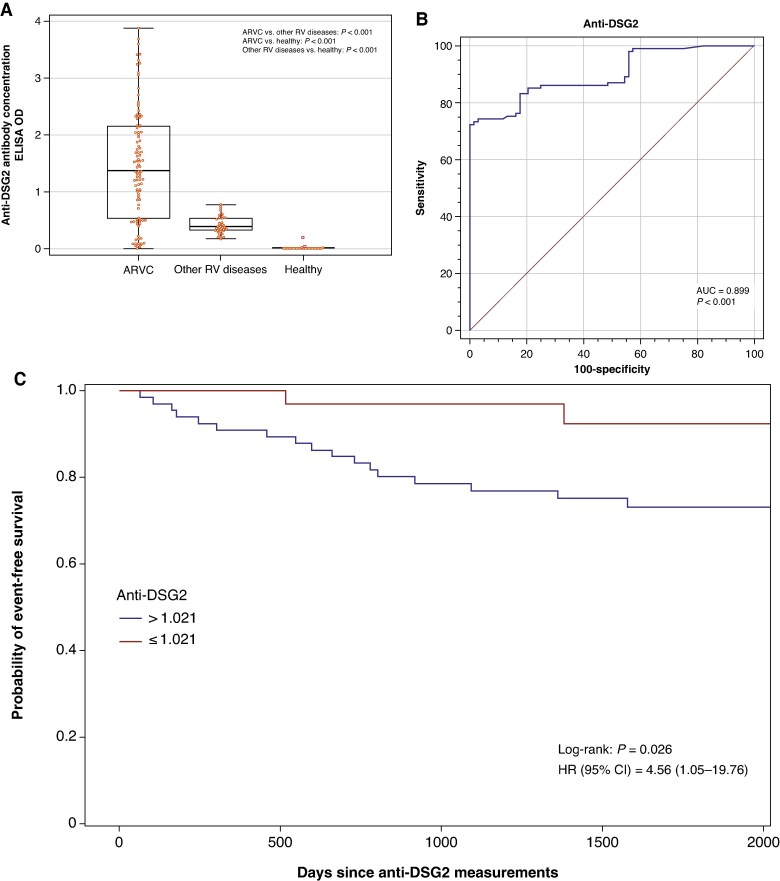
Diagnostic and prognostic performance of anti-DSG2 antibodies in ARVC. (*A*) Distribution of anti-DSG2 antibody levels measured by ELISA (OD) in patients with ARVC, non-ARVC right ventricular cardiac conditions (Ebstein anomaly and Eisenmenger syndrome), and healthy controls. Anti-DSG2 levels were significantly higher in ARVC compared with both control groups (*P* < 0.001 for all comparisons). (*B*) ROC curve demonstrating diagnostic performance of anti-DSG2 antibodies for ARVC. The optimal diagnostic cut-off (OD > 0.774) yielded 72.3% sensitivity and 100% specificity (AUC 0.823). (*C*) Kaplan–Meier survival analysis for the composite endpoint of cardiac death or heart transplantation stratified by anti-DSG2 antibody level. Patients with antibody levels above the prognostic threshold (OD > 1.021) demonstrated significantly reduced event-free survival (hazard ratio 4.56, *P* = 0.026).

Anti-DSG2 antibody levels were significantly higher in patients with ARVC compared with both control groups [median OD 1.36 (0.54–2.15) vs. 0.39 (0.32–0.54) in non-ARVC right ventricular disease and 0.004 (0.002–0.008) in healthy individuals; *P* < 0.001 for all comparisons; *Figure 1A*]. Patients with Ebstein anomaly or Eisenmenger syndrome demonstrated modest antibody elevation relative to healthy controls but substantially lower levels than those observed in ARVC.

Receiver operating characteristic analysis identified an optimal diagnostic cut-off of OD > 0.774, yielding 72.3% sensitivity and 100% specificity for ARVC [area under the curve (AUC) = 0.823; *Figure 1B*]. Using this threshold, 73 of 101 ARVC patients (72.3%) were anti-DSG2 positive, including 74.6% of gene-positive patients and 69.0% of gene-elusive patients. Genetic testing alone identified 59 of 101 patients (58%), antibody testing alone identified 73 (72.3%), and combined biomarker positivity was achieved in 88 of 101 patients (87.1%), representing an incremental diagnostic yield of ∼29% over genetic testing alone.

During a mean follow-up of 3.9 ± 1.6 years, 19 patients reached the primary endpoint of cardiac death or heart transplantation. The diagnostic antibody cut-off was not predictive of outcomes. A higher threshold of OD > 1.021 was associated with an increased risk of cardiac death or heart transplantation on Cox proportional hazards analysis (hazard ratio 4.56, *P* = 0.026; *Figure 1C*). Anti-DSG2 antibody levels were not associated with major arrhythmic events.

## Discussion

This study demonstrates that anti-DSG2 antibodies have promising diagnostic and prognostic utility in ARVC, with a high specificity diagnostic cut-off and meaningful incremental yield over genetic testing alone. Combined antibody and genetic testing identified 87% of the cohort vs. 58% with genetics alone, underscoring the complementary value of serologic assessment, particularly in gene-elusive cases, where conventional diagnostic tools are often insufficient.

These findings are consistent with prior studies reporting that anti-DSG2 antibodies are largely absent in healthy individuals and uncommon in other cardiomyopathies.^7^ Genetic disruption of desmosomal proteins may expose cryptic DSG2 epitopes at the intercalated disc, triggering an autoimmune response and antibody production.^7,8^ Anti-DSG2 antibodies may also impair cell-to-cell adhesion and gap junction integrity, promoting myocardial remodelling and electrical instability.^6,9^ Prior work from our group has reported anti-DSG2 antibody positivity in inflammatory cardiomyopathies, including myocarditis and dilated cardiomyopathy, suggesting shared immune-mediated mechanisms.^5^ Elevated anti-heart and anti-intercalated disc antibodies have similarly been described in ARVC patients and their relatives, further supporting autoimmune contributions to disease pathogenesis.^9,10^ The modest antibody elevation observed in Ebstein anomaly and Eisenmenger syndrome may reflect RV volume or pressure overload causing secondary disruption of intercellular junctions; levels remained substantially lower than in ARVC, supporting the relative specificity of this biomarker for ARVC-related desmosomal pathology.

The association between higher antibody levels and cardiac death or heart transplantation, but not major arrhythmic events, suggests that anti-DSG2 antibodies may reflect progressive myocardial dysfunction. This may identify patients requiring more intensive monitoring or earlier consideration of advanced heart failure therapies.

Limitations include the single-centre design and absence of longitudinal antibody measurements. As comparisons were limited to right ventricular disease controls, diagnostic specificity relative to other non-ischaemic cardiomyopathies more commonly entering the ARVC remains to be established. Larger studies are needed to validate prognostic thresholds and define clinical integration.

## Conclusions

Anti-DSG2 antibodies are elevated in patients with ARVC compared with healthy individuals and other right ventricular diseases, supporting their potential role as a disease-specific biomarker. Higher antibody levels identify patients at increased risk of cardiac death or heart transplantation. Integration of anti-DSG2 testing with clinical and genetic evaluation may improve diagnostic accuracy and risk stratification in ARVC.

## Data Availability

All data are incorporated into the article and its online supplementary material.
